# Effect of the toothbrush tuft arrangement and bristle stiffness on the abrasive dentin wear

**DOI:** 10.1038/s41598-022-04884-x

**Published:** 2022-01-17

**Authors:** Blend Hamza, Maria Niedzwiecki, Philipp Körner, Thomas Attin, Florian J. Wegehaupt

**Affiliations:** 1 Clinic of Orthodontics and Pediatric Dentistry, Center of Dental Medicine, University of Zurich, Plattenstrasse 11, 8032 Zürich, Switzerland; 2 Clinic of Conservative and Preventive Dentistry, Center of Dental Medicine, University of Zurich, Zurich, Switzerland

**Keywords:** Risk factors, Preventive dentistry, Special care dentistry

## Abstract

The geometrical properties of toothbrushes play a role in developing abrasive tooth wear and non-carious cervical lesions. This study investigated the interplay between the toothbrush tuft arrangement (crossed vs. parallel) and bristle stiffness (soft vs. medium) on the abrasive dentin wear using three slurries with different levels of abrasivity (RDA: 67, 121 and 174). Twelve groups of bovine dentin samples (n = 20) were brushed with a combination of the aforementioned variables. Abrasive dentin wear was recorded with a profilometer and the resulting abrasive wear of each group was calculated and compared with each other using two-way ANOVA and pairwise tests. Toothbrushes with parallel tuft arrangement caused statistically significantly higher dentin wear compared to crossed tuft arrangement, regardless of the abrasivity level of the used slurry and the bristle stiffness. Soft crossed tuft toothbrushes caused statistically significantly higher abrasive dentin wear than medium crossed tuft toothbrushes, while soft and medium parallel tuft toothbrushes caused the same amounts of dentin wear, regardless of the RDA value of the used slurry. These results could be helpful for dentists and dental hygienists when advising patients. Crossed tuft toothbrushes could be a less-abrasive choice in comparison to parallel tuft toothbrushes.

## Introduction

Toothbrushes belong to daily routine as the most widely used tool for oral hygiene^1^. During the present and past centuries, toothbrushes have undergone remarkable changes in their design, especially at the toothbrush head (e.g., number of tufts, brushing plane, bristle stiffness and diameter)^2^. Many of these design changes perused one goal: to achieve better cleaning effect^3^.

It is well known that toothbrushing not only removes dental plaque, but could also remove parts of the sound tooth hard tissue (abrasive enamel or dentin wear)^4^. This abrasive wear was classified as an important factor in the development of non-carious cervical lesions (NCCL) in many studies^5–10^.

Several factors were deemed responsible for the development of the abrasive wear (e.g., brushing force and frequency, abrasivity of the used toothpaste, toothbrush bristle design)^11^. Basically, changing any of these factors would eventually change the resulting abrasive wear. For instance, a recent study reported that soft and medium bristles caused comparable abrasive dentin wear until 3-N brushing force was reached. Soft bristles, however, caused statistically significantly less abrasive dentin wear at 4 N^12^.

While plenty of studies investigated the effect of different tuft designs on the cleaning efficacy, studies investigating the same effect on the resulting abrasive wear are scarce^13–16^. It could be speculated that different geometrical properties of the toothbrush would alter the way abrasives are activated on the tooth surface, and eventually alter the resulting abrasive wear. Therefore, this study was conducted to investigate the effect of two different tuft designs (parallel vs. crossed) and two different bristle stiffness (soft vs. medium) on the abrasive dentin wear using three abrasive slurries with different relative dentin abrasion (RDA) values (67, 121 and 174).

## Material and methods

For this study, 240 dentin samples from bovine lower permanent incisors were prepared. The bovine sacrifice was carried out for food processing and had no relation with this study. Each sample (diameter = 3 mm) was drilled out from the root using a diamond trephine mill under water-cooling (Proxxon, Föhren, Germany). The samples were then embedded in acrylic resin (Paladur, Heraeus Kulzer, Hanau, Germany), which was allowed to set in a laboratory polymerizer (Palamat elite, Heraeus Kulzer, Germany) at 55 °C and 2 bar for 10 min. The samples were then ground at 5 N pressure load and 80 rpm in a grinding machine (Tegramin-30, Stuers, Birmensdorf, Switzerland) with 2000- and then 4000-grit carborundum paper for 20 and 30 s, respectively. Additional details regarding the preparation of the samples can be obtained from an earlier study^17^. The samples were randomly allocated into twelve groups (n = 20). Baseline profiles were recorded for all samples using a contact profilometer (MFW803, Perthometer S2; Mahr, Göttingen, Germany) under wet conditions.

A bushing sequence (120 strokes/min, at 2.5 N, and for 25 min) was then carried out according to each group specification as follows: Groups 1–3 were brushed with a soft toothbrush (crossed tuft design) using abrasive slurries with 67, 121 and 174 RDA value, respectively. Groups 4–6 were brushed with a soft toothbrush (parallel tuft design), groups 7–9 were brushed with a medium toothbrush (crossed tuft design) and groups 10–12 were brushed with a medium toothbrush (parallel tuft design) with the same abrasive slurries, respectively.

**Figure 1:**
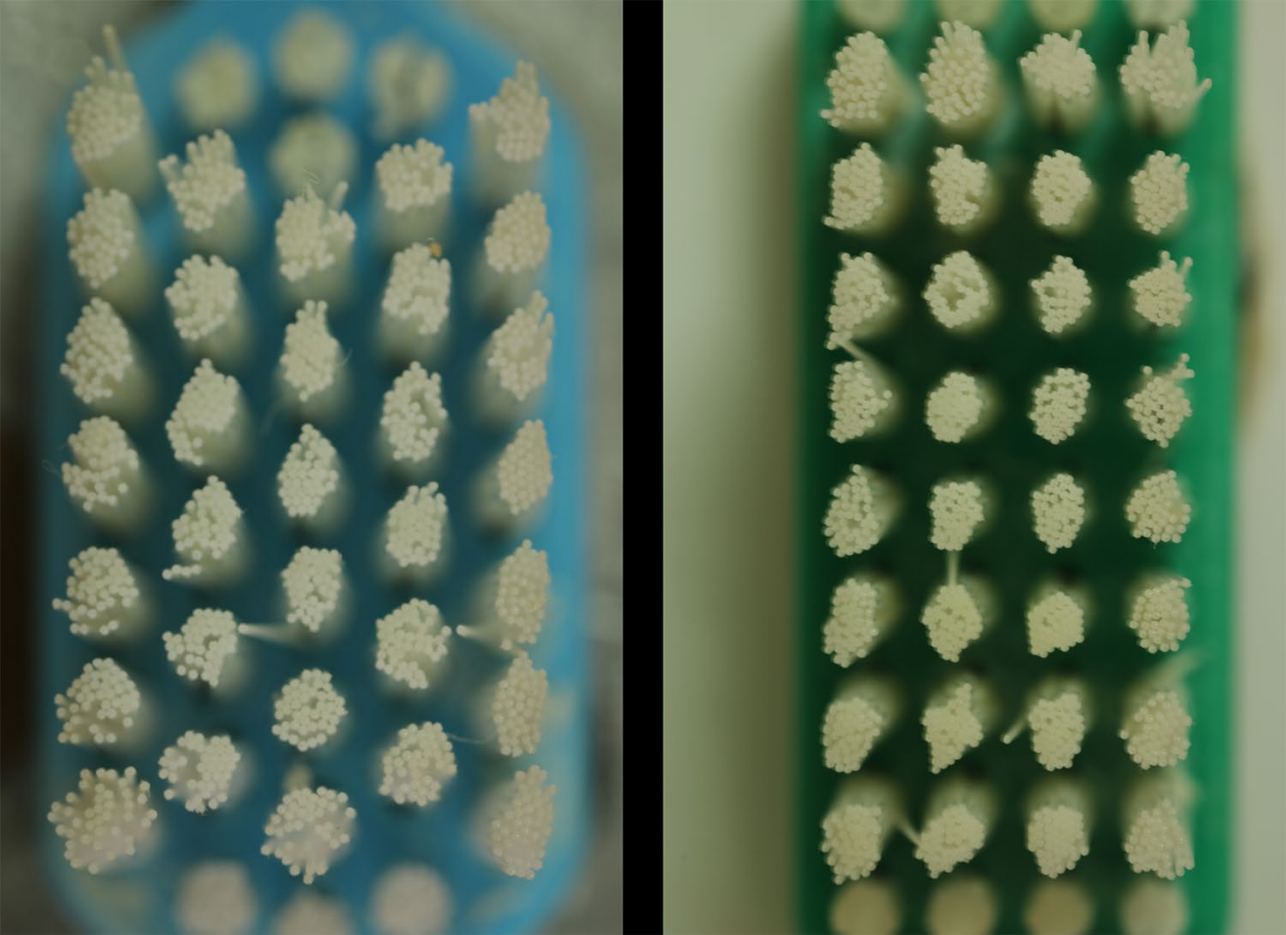
Top view pictures of the crossed tuft (left) and parallel tuft (right) toothbrushes (both medium bristle stiffness. The soft bristle toothbrushes had the same geometric profile).

M43 (parallel tuft design, soft and medium) and M39 (crossed tuft design, soft and medium) toothbrushes were used in this study (Esro, Thalwil, Switzerland). Apart from tuft design (crossed vs. parallel) and bristle length (soft vs. medium), all tested toothbrushes had the same bristle properties. In order to obtain the same number of tufts per toothbrush, some tufts were cut from the heel and toe of both toothbrushes (parallel tuft: four tufts from the heel and seven tufts from the toe, crossed tuft: four tufts from the heel and three tufts from the toe). Table 1 shows the properties of the used toothbrushes and Fig. 1 shows a top view photo of them.

**Table 1:** Properties of the tested toothbrushes.

Toothbrush type	Soft crossed (M39) and parallel (M43) tuft design	Medium crossed (M39) and parallel (M43) tuft design
Bristle diameter	0.2 mm	0.2 mm
Bristle length	12 mm	10.5 mm
Material	Polyamide	Polyamide
Tip configuration	End-rounded	End-rounded
Number of tufts	32	32
Number of bristles per tuft	40 ± 4	40 ± 4

To prepare the abrasive slurry, 90 g of the abrasive Zeodent® 113 (RDA = 67) or Zeodent® 103 (RDA = 174) (Evonik industries, Hanau-Wolfgang, Germany) were mixed with 450 g hydroxyethylcellulose-glycerine-mix and 0.45 g of a silicon antifoaming agent. For the abrasive slurry with the RDA value of 121, 45 g of Zeodent® 103 and 45 g of Zeodent® 113 abrasives were used. After the brushing sequence, final profiles were recorded according to previously well-established protocol under wet conditions^18^. Table 2 summarizes the study protocol.

**Table 2:**
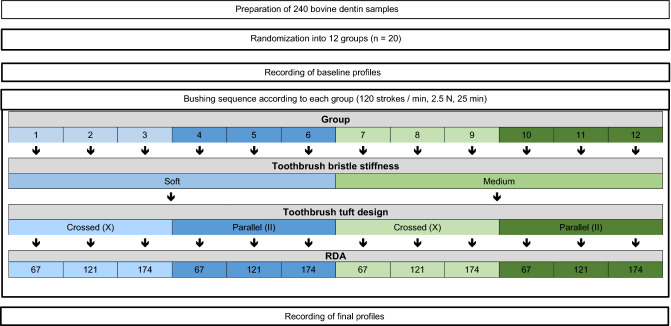
Study design.

### Statistical analysis

Median and interquartile range (IQR) of the abrasive dentin wear for each tested group were calculated. Two-way ANOVA test was applied to investigate whether significant differences between the groups were present. Pairwise contrasts between the groups brushed with the same abrasive slurry (same RDA value) were tested and the *p* value was corrected according to Tukey method for multiple tests. Significance level was set at 0.05 and data was processed with R software (The R Foundation for Statistical Computing; Vienna, Austria; www.R-project.org).

## Results

Two-way ANOVA test revealed a significant influence of each variance in this study (RDA value (*p* < 0.001), tuft design (*p* < 0.001), and bristle stiffness (*p* < 0.01)).

Within the groups brushed with the 67-RDA abrasive slurry, the median (IQR) abrasive dentin wear according to the properties (bristle stiffness and tuft design) was calculated as follows: Soft crossed = 4.85 µm (2.6), soft parallel = 8.50 µm (1.0), medium crossed = 4.40 µm (1.6) and medium parallel = 9.05 µm (1.1).

For groups brushed with the 121-RDA abrasive slurry, the median (IQR) abrasive dentin wear according to the properties (bristle stiffness and tuft design) was calculated as follows: Soft crossed = 6.20 µm (1.6), soft parallel = 15.75 µm (3.5), medium crossed = 4.75 µm (2.6) and medium parallel = 13.30 µm (3.5).

Using the 174-RDA abrasive slurry, the median (IQR) abrasive dentin wear according to the properties (bristle stiffness and tuft design) was calculated as follows: Soft crossed = 8.10 µm (2.6), soft parallel = 17.00 µm (5.6), medium crossed = 5.10 µm (2.7) and medium parallel = 16.95 µm (2.4).

**Figure 2:**
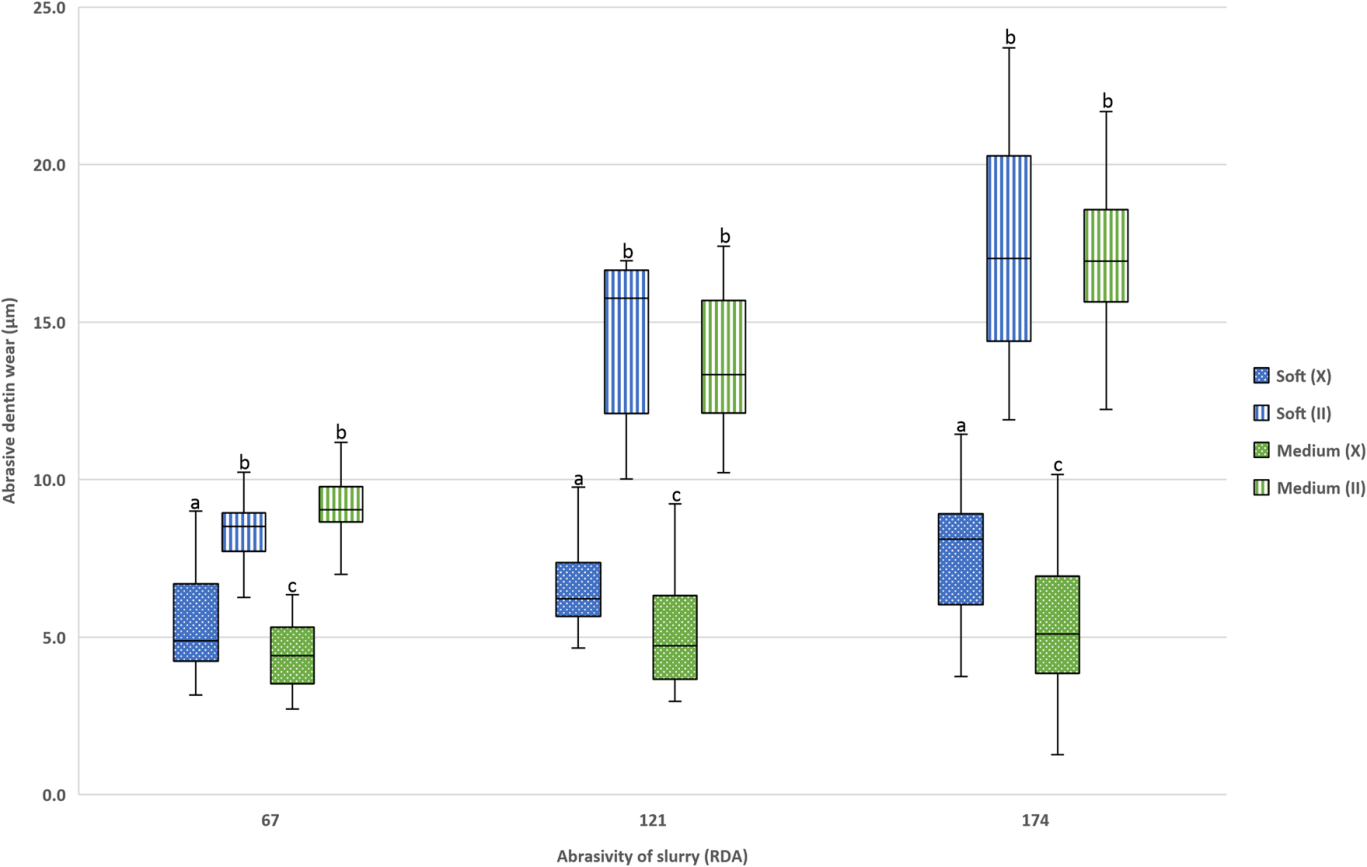
Abrasive dentin wear (median + interquartile range, IQR = whiskers) in the experimental groups [crossed tuft design (X), parallel tuft design (II)]. Same letters indicate no statistically significant difference within the respective abrasive slurry (same RDA value).

Within all above-mentioned groups, all differences were statistically significant (*p* ≤ 0.0001) except between the toothbrushes with parallel tuft design (soft vs. medium, *p* = 0.98). Figure 2 shows the abrasive dentin wear for each group.

## Discussion

Toothbrushes are now designed based on scientific data. The effect of different tuft designs and bristle stiffness on the abrasive dentin wear is yet to be thoroughly comprehended. This study compares the abrasive dentin wear resulting from soft and medium bristles in crossed and parallel tuft designs, using abrasive slurries with different abrasivity values (RDA = 67, 121 and 174). The possible differences in the abrasive wear could be helpful for dentists and dental hygienists in terms of advising patients suffering from abrasive dentin wear and NCCL.

Due to its larger surface, more samples can be obtained from a single bovine tooth compared to a human one. The fact that bovine teeth are an appropriate alternative to human teeth in abrasion studies has been asserted in a previous study^19^. Samples were brushed at 2.5-N brushing force for 25 min in this study. 2.5 N was reported by Ganss et al.^20^ as the in-vivo mean brushing force applied by uninstructed patients using a manual toothbrush (2.3 ± 0.7), and is also within the range of brushing forces used by most abrasion studies (2–3 N)^21^. The brushing time of 25-min reflects about seven-month brushing time clinically, assuming that the patients brush twice daily for 2 min^22^. In this regard, it could be argued that 25 min of continuous brushing does not resemble the clinical situation, where the teeth are brushed intermittently. Krol et al.^23^ investigated this matter and reported that the abrasive dentin wear resulting from continuous 25-min brushing time was similar to the abrasive dentin wear resulting from intermittent brushing (25 brushing cycles, 1 min each), regardless of the RDA value of the used slurry. Nevertheless, the fact that in-vitro brushing cycles do not allow the formation of a salivary pellicle could be considered as a shortcoming, as the pellicle could reduce the resulting abrasive wear clinically^24^. As mentioned above, the here used 25 min brushing time applied with the toothbrush corresponds to about 7 month brushing time in vivo. However, under clinical situations, it is usually recommended to change toothbrushes after 3 months. This might raise some questions on the relevance of the method vs consumer/patient use. The here used brushing time is kind of a standard and regularly used in testing abrasive dentin wear in vitro^23,25,26^. Furthermore, it's a worse-case scenario which might be reached under clinical conditions much later. Regardless, as there is a large variation across consumers and within consumers across their dentition, a certain type of “standardization” has to be chosen.

The different bristle length gave the tested toothbrushes their soft “longer bristles” or medium “shorter bristles” property. Other than the tuft arrangement, all other bristle properties of all tested toothbrushes remained the same as shown in Table 1. These uniform properties allowed the investigation of the pure effect of the bristle stiffness and tuft arrangement on the resulting abrasive wear, and could be viewed as a strength of this study.

Parallel tuft toothbrushes always caused statistically significantly higher abrasive dentin wear compared to crossed tuft toothbrushes, regardless of the RDA value of the applied slurry and the bristle stiffness. This might be explained by the fact that the crossed tufts geometrically leads to fewer spaces between the tufts in comparison to the parallel tufts. The dense proximity of the tufts in the crossed tuft design toothbrush could have led the abrasive particles to be trapped tight within the tufts—at least in the x-axis—and consequently prevented some amount of them from being entrapped underneath the bristles. On the other hand, parallel tuft toothbrushes could have allowed the abrasive particles to “travel” between the tufts—in both x- and y-axis—, giving the chance for more of them to be entrapped underneath the bristles and consequently be rubbed on the dentin surface causing some abrasive dentin wear (see Fig. 1). In this regard, it was interesting to observe that the higher the RDA value of the slurry, the higher was the difference of the resulting abrasive wear caused by parallel tuft toothbrushes compared to crossed tuft toothbrushes. This point cannot be explained by assuming that more abrasive particles were available in the slurries with high RDA values, as all abrasive slurries contained the same amount of abrasives to avoid different viscosities, which might have—falsely—altered the resulting abrasivity.

Soft crossed tuft toothbrushes caused statistically significantly higher abrasive dentin wear than medium crossed tuft toothbrushes, while soft and medium parallel tuft toothbrushes caused the same amounts of abrasive wear, regardless of the RDA value of the employed abrasive slurry. These two observations contribute to the current uncertainty regarding the influence of bristle stiffness on the abrasive dentin wear. In a recent study by Hamza et al.^12^, where the same soft- and medium parallel tuft toothbrushes were used as in this study (M43), same amounts of abrasive dentin wear were also observed between the two kinds of bristle stiffness at 2- and 3-N brushing force. On the other hand, another study by Bizhang et al.^27^ reported that soft bristle toothbrush caused statistically significantly higher abrasive dentin wear than medium bristle toothbrush, which is actually in agreement with the present findings with the crossed tuft design. However, the toothbrushes used in the study of Bizhang et al.^27^ had two-level bristle design, different number of bristles per tuft, and no information was given whether the tuft design was parallel or crossed. Thus, it might not be accurate to compare the observations of both studies with each other. In contrast to the present study, Turssi et al.^28^ found medium and hard bristle toothbrushes to cause statistically significantly higher abrasive dentin wear than soft bristle toothbrushes at 2-N brushing force using abrasive slurries with medium and high abrasivities. Again, the used toothbrushes had different bristle diameter and number of bristles per toothbrush, which might have influenced the results. As the bristle properties in all tested toothbrushes in the present study had the same properties—other than stiffness and tuft arrangement—, it could be considered that the present results give a more realistic approach to the effect of the toothbrush tuft design and bristle stiffness on the abrasive dentin wear.

It is worth mentioning that the soft bristles in the previous study of Hamza et al.^12^ caused statistically significantly less abrasive dentin wear than the medium bristles when the brushing force reached 4 N. This was attributed to the fact that the soft bristles deflected much more than the medium bristles at such brushing force and dragged less abrasive particles on the samples’ surface. Considering this finding, it could theoretically be assumed that crossed tuft design would rather prevent the bristles from getting deflected in comparison to the parallel tuft design, and therefore cause more abrasive wear. This assumption might also explain the fact that soft crossed tuft toothbrushes caused statistically significantly more abrasive wear than medium crossed tuft toothbrushes when—only—a 2.5-N brushing force was applied in the present study. It could be interesting to investigate how tuft design interacts with increasing brushing forces in future studies.

Based on the present study and within its limits, it could be concluded that toothbrushes with crossed tufts cause less abrasive dentin wear than those with parallel tufts. This conclusion should be kept in mind along with other abrasive wear contributing factors when advising patients with signs of abrasive dentin wear.

## Data Availability

The datasets generated during and/or analyzed during the current study are available from the corresponding author on reasonable request.
